# Perinatal Malnutrition Leads to Sexually Dimorphic Behavioral Responses with Associated Epigenetic Changes in the Mouse Brain

**DOI:** 10.1038/s41598-017-10803-2

**Published:** 2017-09-11

**Authors:** Daniel Nätt, Riccardo Barchiesi, Josef Murad, Jian Feng, Eric J. Nestler, Frances A. Champagne, Annika Thorsell

**Affiliations:** 1 Center for Social and Affective Neuroscience, Department of Clinical and Experimental Medicine, Linköping University, Linköping, Sweden; 2 Department of Psychology, Columbia University, New York, NY USA; 3 Department of Biological Science, Florida State University, Tallahassee, FL USA; 4 Fishberg Department of Neuroscience and Friedman Brain Institute, Icahn School of Medicine at Mount Sinai, New York, NY USA

## Abstract

Childhood malnutrition is a risk factor for mental disorders, such as major depression and anxiety. Evidence shows that similar early life adversities induce sex-dependent epigenetic reprogramming. However, little is known about how genes are specifically affected by early malnutrition and the implications for males and females respectively. One relevant target is neuropeptide Y (NPY), which regulates both stress and food-intake. We studied maternal low protein diet (LPD) during pregnancy/lactation in mice. Male, but not female, offspring of LPD mothers consistently displayed anxiety- and depression-like behaviors under acute stress. Transcriptome-wide analysis of the effects of acute stress in the amygdala, revealed a list of transcription factors affected by either sex or perinatal LPD. Among these immediate early genes (IEG), members of the *Early growth response* family (*Egr1*/2/4) were consistently upregulated by perinatal LPD in both sexes. EGR1 also bound the *NPY receptor Y1* gene (*Npy1r*), which co-occurred with sex-specific effects of perinatal LPD on both *Npy1r* DNA-methylation and gene transcription. Our proposed pathway connecting early malnutrition, sex-independent regulatory changes in *Egr1*, and sex-specific epigenetic reprogramming of its effector gene, *Npy1r*, represents the first molecular evidence of how early life risk factors may generate sex-specific epigenetic effects relevant for mental disorders.

## Introduction

Maternal malnutrition during pregnancy and lactation is a global problem, affecting millions of women and children world-wide, especially in low-income countries^1, 2^. This type of early adversity influences the offspring later in life^3–5^. Brain development seems particularly sensitive, where early life malnutrition leads to structural and molecular alterations^6^ which affect personality traits and mental health^7, 8^, including the risk to develop major depression^9^. As a form of malnutrition, protein insufficiency in early life has primarily been studied in relation to metabolic and cardiovascular diseases in adult offspring^10–12^. However, associations observed between early life protein insufficiency and behavioral changes suggests effects on neural function^13, 14^. The molecular events underlying alterations following a low protein diet is poorly understood, although epigenetic reprogramming in specific brain regions that intersect nutritional and behavioral regulation, such as the hypothalamus, has been implicated^11, 15^.

Neuropeptide Y (NPY)^16, 17^, is a relevant candidate for the neurodevelopmental effects following prenatal malnutrition since it regulates both feeding and anxiety. NPY’s role in controlling feeding behavior involves regulating energy homeostasis from within the hypothalamus^18, 19^. This is mediated primarily via Y1 and Y5-receptors, where the Y1 receptor mediates the anabolic effects following elevated NPY levels in this brain region^19–22^. Anxiety is controlled primarily via actions within the amygdala^21, 23–25^, where an interplay between postsynaptic Y1 and presynaptic Y2 receptors in specific sub-nuclei is believed to control the anxiolytic effects of NPY^25^. NPY neuronal projections from hypothalamus to amygdala have also been described^26, 27^, further strengthening its relevance in both feeding and anxiety. However, little is known about how NPY related actions in the hypothalamus specifically affects the anxiolytic actions in amygdala.

Expression of NPY and its receptors are regulated via several stress-related transcription factors, such as the glucocorticoid receptor^28, 29^ and immediate early genes (IEGs)^30^. IEGs are biologically conserved genes that rapidly and transiently change their expression, independent of *de novo* protein synthesis, in response to intra- and extracellular stimuli^31, 32^, of which some may be considered exogenous stressors such as physical restraint, food deprivation and exposure to drugs of abuse^33–36^. Many IEGs work as transcription factors by binding specific regulatory regions of target genes, which then carry out specific effector functions. Several IEGs are known to regulate networks of effector genes involved in neural activation and plasticity^37^. Among these are the Fos-family transcription factors (e.g. *cFos*, *Fosb*), as well as the early growth response elements (*Egr1*, 2*, 3, 4*). EGR1 (also Zif268/krox24/NGFI-A)^38^ is of particular interest since it has been implicated in a critical mechanism linking early life social adversity with adult neuropsychiatric outcomes, through interplay with epigenetic mechanisms (such as DNA-methylation) at the binding sites of its effector genes in relevant brain regions^39, 40^. Thus, studying the epigenetic landscapes at relevant IEG effector genes, in brain regions known to control anxiety (e.g. amygdala and hypothalamus), may be an effective strategy for disentangling the molecular events involved in the neurodevelopmental effects following perinatal malnutrition.

Here, we studied the effect of early life malnutrition on behavioral and gene regulatory responses following acute stress (forced swimming) in adult mice. We hypothesize that a low protein diet (LPD) during pregnancy and lactation would induce anxiety- and depression-like behaviors in adult offspring, and that these behaviors would be reflected in the regulatory interactions between the IEG and NPY systems in hypothalamus and amygdala. To account for sex-dependent effects, we included both male and female offspring in the study.

## Results

### Maternal low protein diet modulates behaviors following acute stress in the offspring

**Figure 1:**
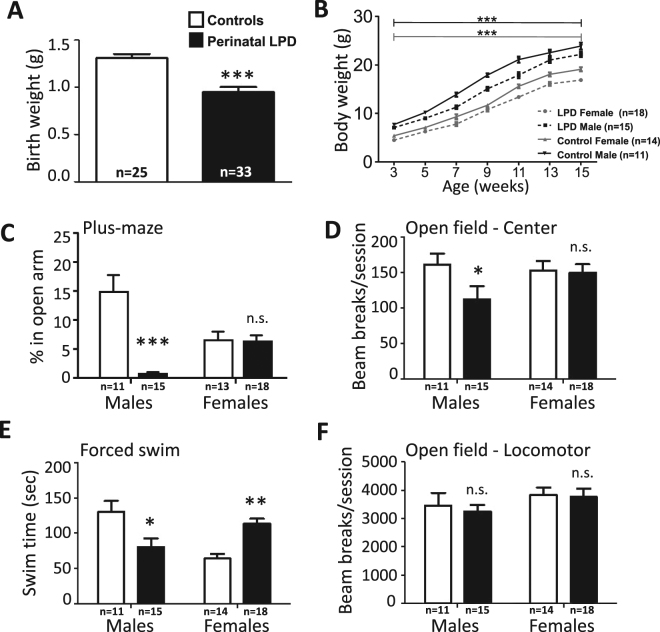
Adult male offspring from dams fed a low protein diet display increased anxiety- and depression- like behavior. Bodyweight at birth was significantly reduced in offspring from LPD dams (**A**), a difference that was maintained throughout adulthood albeit weight gain was not significantly different between groups (**B**). Anxiety-like behavior was measured on the elevated plus-maze. Open-arm exploration in adult male offspring from LPD dams was significantly reduced compared to control offspring, indicating an anxiety-like phenotype (**C**). Perinatal LPD had no effect on female anxiety, however, females generally displayed lower exploration of the open arms compared to male controls. These profiles were replicated using the open-field as an alternative assessment of anxiety, measured as activity in the center of the arena (**D**). In the forced swim test, a model sensitive to anti-depressant drugs, increased immobility was seen in adult male offspring from LPD dams, indicating a depression-like phenotype, while results in females were inversed, indicating a protective effect by perinatal LPD (**E**). General locomotor behavior, as measured by total activity in the open-field, showed no difference between sexes or perinatal LPD treatment (**F**). Asterisks in (**C**–**F**) represent results of Newman-Keul post-hoc tests in full factorial general linear models with Sex and Perinatal LPD as independent factors. Supporting results, see main text, Tables S1 and S2, Figs S1 and S2.

Maternal LPD did not affect the number of successful pregnancies or the number of pups born per litter, nor were there significant differences in dam body-weight at birth and weaning (Table S1). However, the birth-weights of pups from dams fed LPD was lower than in dams fed a control diet (Fig. 1A). Pup weight gain was not significantly affected by maternal diet. However, a significant reduction in body-weight remained throughout the study (Fig. 1B; Time effect: F[6, 324] = 121.8; p < 0.0001; Group effect (diet and sex): F[3, 54] = 24.85; p < 0.0001; Time x Group effect: F[18, 324] = 5.134; p < 0.0001).

There were substantial sex-differences in adult stress-related behaviors in the offspring, which was dependent on perinatal LPD exposures. Male offspring of LPD dams displayed a pronounced anxiety profile on the elevated plus-maze (Fig. 1C; Perinatal LPD: F[1, 53] = 20.19; p < 0.0001, Sex: F[1, 53] = 0.59; n.s.; Perinatal LPD x Sex: F[1, 53] = 21.07; p < 0.0001), while there was no effect in female offspring. This finding was mirrored in the open-field, where activity in the center was significantly reduced in male offspring from LPD dams compared to control offspring, while there was no effect in females exposed to perinatal LPD (Fig. 1D).

The forced swim test assesses responses to acute stress, and animal mobility during the test correlates with antidepressant-like endpoints. Male offspring of LPD dams displayed increased immobility in the forced swim test, interpreted as increased depression-like behavior, compared to control males (Fig. 1E). Female offspring, on the contrary, displayed increased mobility (antidepressant-like behavior) following perinatal LPD compared to controls (Fig. 1E; Perinatal LPD: F[1, 54] = 0.2; p = n.s., Sex: F[1, 54] = 2.93; p < 0.1; Perinatal LPD x Sex: F[1, 54] = 26.3; p < 0.0001).

Control behaviors such as overall locomotion in the open field (Fig. 1F), as well as pain thresholds measured by tail flick (Fig. S1) and hot plate behavior (Fig. S2), were not significantly different between perinatal diet-groups. Adult health assessments (behavioral and physical)^41^ showed no effect of perinatal LPD in either of the sexes, except for perinatal LPD males having different response to a novel environment (Table S2).

### IEG expression following acute forced swim stress in amygdala

**Figure 2:**
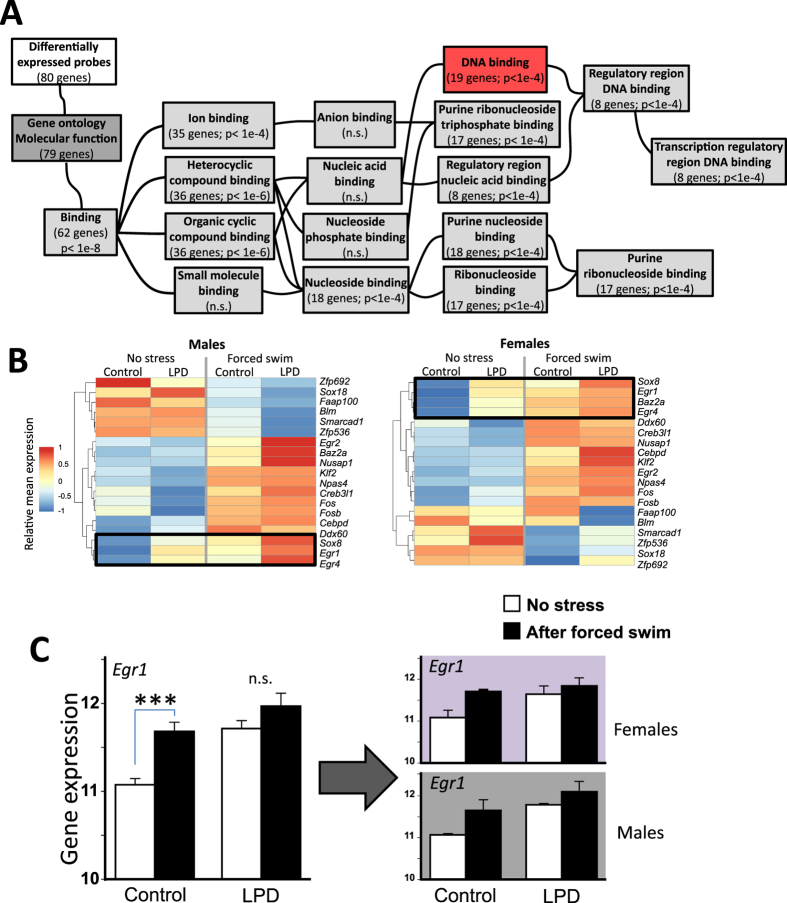
Maternal low protein diet affects stress-dependent gene expression of *Egr1* and other immediate early transcription factors in amygdala. A large proportion of the validated transcripts shown to be affected by 15 min acute forced swim came from genes involved in DNA binding (GO: 0003677), thus potentially being IEG transcription factors (**A**). Among these, *Egr1*/*2* and *Sox8*, shared the same clade in a hierarchical cluster analysis, suggesting co-regulation (**B**). The effect of Forced Swim and Perinatal LPD on *Egr1* was statistically confirmed (**C**) using a two-way factorial Generalized linear model (factors: Sex | Acute stress | Perinatal LPD). ***represent p < 0.001 in pairwise comparisons within each perinatal LPD groups after Bonferroni correction. In the original microarray experiment, 32 adult animals were pooled 2 by 2, and hybridized to 16 microarrays, equally distributed between Sex (males vs. females), Acute stress (forced swim vs. no stress) and Perinatal LPD (maternal LPD vs regular diet).

To identify the gene regulatory changes underlying the sex-specific behavioral differences following perinatal LPD, we screened for transcriptomic changes in the amygdala using the Agilent Mouse Exon 4 × 180 K Microarrays platform. Knowing that perinatal LPD dramatically affected the behavioral response to acute stress, and that acute stressors previously have been associated with changes in immediate early gene expression in the amygdala^42–44^, we limited our screening to the transcriptional effects of perinatal LPD in this brain region following 15 min acute forced swimming. This dramatically decreased the risk to make false negative errors following adjustments for multiple testing. After an extensive validation procedure, involving full technical replication of all the male samples on separate microarrays (see Methods and Fig. S3), we identified 104 microarray probes (80 genes) that were consistently affected by 15 min forced swimming (Table S3). A substantial number of these genes were also affected by other types of acute stress (foot-shock and restraint) in a dataset^42^ generated using a different microarray platform (Table S4). A gene ontology analysis showed that 24% of the identified genes were binding DNA, thus potentially being IEG transcription factors (Fig. 2A).

### Transcription of the Egr family was robustly affected by maternal LPD in amygdala

Most of the IEG transcription factor genes were upregulated in amygdala by acute stress (Table 1). Among the identified genes were well-known IEGs such as *Fos*, *FosB* and *Npas4*. More relevant for the present study, the early growth response family members, *Egr1* and *Egr4* (and in some aspects *Egr*2), were strongly affected by perinatal LPD, and clustered together with *Sox8* as if they were co-regulated (Fig. 2B). Of these genes, *Egr1* was particularly robust, showing highly similar results also in the male validation experiment (Fig. S4). Closer examination showed that *Egr1* expression was similarly affected by perinatal LPD in both sexes, and that the acute stress effect was mainly seen in the controls, since perinatal LPD exposed animals had increased baseline expression that reached the levels of control animals exposed to forced swimming (Fig. 2C; Generalized Linear Model; Perinatal LPD: χ^2^ = 30.0, p < 0.0001; Forced Swim: χ^2^ = 26.0, p < 0.0001; Perinatal LPD x Forced Swim: χ^2^ = 4.3, p < 0.05). To better understand the specificity of these effects, we compared our results with three microarray datasets covering the effects of other chronic stressors in the amygdala of adult mice; fear conditioning^42^, zinc restricted diet^45^, and chronic alcohol exposure^46^. Results showed that, while other IEG transcription factors were affected across stressors, only fear conditioning in an anxiety prone mouse strain^42^ induced similar changes in baseline levels of the *Egr* family genes, as was observed in Perinatal LPD offspring (Fig. S5).

**Table 1:** Immediate early transcription factor expression in amygdala identified in microarray experiment.

Transcript	SEX		LPD		STRESS*		Interactions	
	Males vs Females		LPD vs Control		Forced swim vs No stress		LPD × SEX	LPD × STRESS
	Direction	p-value	Direction	p-value	Direction	p-value	p-value	p-value
*Faap100*	Down	<0.05^a^			Down	<0.001		
*Baz2a*			Up	<0.05^e^	Up	<0.001		
*Blm*			Down	<0.01^f^	Down	<0.001		
*Cebpd*	Down	<0.001^b^	Up	<0.05^g^	Up	<0.001		
*Creb3l1*					Up	<0.001		
*Ddx60*					Up	<0.001		
*Egr1*			**Up**	**<0.001** ^**h**^	Up	<0.001		**<0.05** ^**q**^
*Egr2*			**Up**	**<0.05** ^**i**^	Up	<0.001		
*Egr4*			**Up**	**<0.001** ^**j**^	Up	<0.001		
*Fos*					Up	<0.001	<0.05^m^	
*Fosb*					Up	<0.001		
*Klf2*					Up	<0.001	<0.05^n^	
*Npas4*					Up	<0.001		
*Nusap1*					Up	<0.001		
*Smarcad1*	Down	<0.001^c^			Down	<0.001	<0.05°	
*Sox18*					Down	<0.001		
*Sox8*	Down	<0.001^d^	Up	<0.001^k^	Up	<0.001		
*Zfp536*					Down	<0.001		
*Zfp692*			Down	<0.05 ^l^	Down	<0.001	<0.01^p^	<0.05^r^

Arrows indicate relative transcriptional changes between groups in the respective comparison (see top row, beneath SEX, LPD, STRESS respectively).

Two-way Generalized linear models were used resulting in these estimated marginal means with standard errors (assuming equal variance) based on RMA normalized microarray intensities:

^a^Males (n = 8): 4.91 + / − 0.1; Females (n = 8): 5.22 + / − 0.1, ^b^Males (n = 8): 8.57 + / − 0.06; Females (n = 8): 8.91 + / − 0.06, ^c^Males (n = 8): 8.23 + / − 0.04; Females (n = 8): 8.46 + / − 0.04, ^d^Males (n = 8): 9.02 + / − 0.03; Females (n = 8): 9.18 + / − 0.03, ^e^LPD (n = 8): 6.24 + / − 0.04; Control (n = 8): 6.11 + / − 0.04, ^f^LPD (n = 8): 4.15 + / − 0.09; Control (n = 8): 4.48 + / − 0.09, ^g^LPD (n = 8): 8.83 + / − 0.06; Control (n = 8): 8.65 + / − 0.06, ^h^LPD (n = 8): 11.84 + / − 0.06; Control (n = 8): 11.38 + / − 0.06, ^i^LPD (n = 8): 8.14 + / − 0.08; Control (n = 8): 7.91 + / − 0.08, ^j^ LPD (n = 8): 11.43 + / − 0.04; Control (n = 8): 11.09 + / − 0.04, 0.04, ^k^LPD (n = 8): 9.22 + / − 0.03; Control (n = 8): 9.00 + / − 0.03, ^l^LPD (n = 8): 5.70 + / − 0.07; Control (n = 8): 5.88 + / − 0.07, ^m^LPD males (n = 4): 6.43 + / − 0.2; Control males (n = 4): 6.64 + / − 0.2; LPD females (n = 4): 6.54 + / − 0.2; Control females (n = 4): 5.80 + / − 0.2, ^n^LPD males (n = 4): 6.68 + / − 0.08; Control males (n = 4): 6.76 + / − 0.08; LPD females (n = 4): 6.99 + / − 0.08; Control females (n = 4): 6.74 + / − 0.08, ^o^LPD males (n = 4): 8.20 + / − 0.05; Control males (n = 4): 8.27 + / − 0.05; LPD females (n = 4): 8.54 + / − 0.05; Control females (n = 4): 8.38 + / − 0.05, ^p^LPD males (n = 4): 5.54 + / − 0.09; Control males (n = 4): 6.00 + / − 0.09; LPD females (n = 4): 5.85 + / − 0.09; Control females (n = 4): 5.75 + / − 0.09, ^q^LPD forced swim (n = 4): 11.97 + / − 0.09; Control forced swim (n = 4): 11.68 + / − 0.09; LPD no stress (n = 4): 11.71 + / − 0.09; Control no stress (n = 4): 11.07 + / − 0.09, ^r^LPD forced swim (n = 4): 5.57 + / − 0.09; Control forced swim (n = 4): 5.57 + / − 0.09; LPD no stress (n = 4): 5.82 + / − 0.09; Control no stress (n = 4): 6.19 + / − 0.09.

*Means for the main effect of acute stress is not shown, since they were acquired by whole genome analysis.

### NPY transcription associates with EGR1 binding in amygdala and hypothalamus

**Figure 3:**
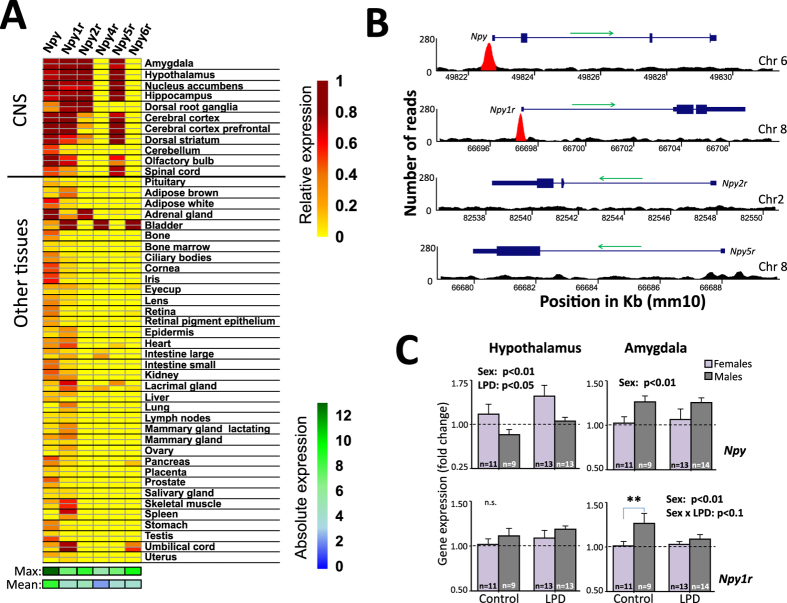
*Npy1r* is expressed in the adult mouse brain, is targeted by EGR1 binding and affected by maternal low protein diet. Three out of five NPY receptor genes are widely expressed together with *Npy* in the mouse brain, including amygdala, hypothalamus, and areas of the striatum (e.g. nucleus accumbens) (**A**). Expression values are represented by microarray probeset duplicates as reported in the BioGPS database: Npy = 1419127_at; Npy1r = 126054_at; Npy2r = 1417489_at; Npy4r = 1422271_at; Npy5r = 1449312_at; Npy6r = 1438086_at. ChIP-seq analysis from a previous experiment targeting the striatum revealed that among the brain specific Npy receptor genes, EGR1 only binds the promoter region of *Npy* and *Npy1r* (**B**). Sex and Perinatal LPD affected *Npy* and *Npy1r* expression differently in amygdala compared to hypothalamus, as measured by qPCR (**C**). Bar graphs show the pairwise sex differences of the generalized linear models first presented in Table 2. In hypothalamus, *Npy* expression was affected by both Sex and Perinatal LPD, where perinatal LPD led to more *Npy* expression regardless of sex, but where females generally experienced more expression than males. No effects were seen on hypothalmic *Npy1r* expression. In amygdala, the Sex effect in *Npy* expression was inversed compared to the Sex effect in hypothalamus. Sex also affected amygdala *Npy1r* expression, but a post-hoc test (Bonferroni; **p < 0.01) indicated that this was mainly driven by a sex difference in controls that was absent in animals exposed to Perinatal LPD.

Since sex had limited effects on *Egr*-family gene expression, which were strongly affected by perinatal LPD, we hypothesized that the sex differences observed in behavior could instead be reflected in the interactions between the *Egr*-family transcription factors and their target effector genes. To limit our analysis, we focused on EGR1 and the NPY system. First, we verified the transcription of *Npy* and its receptors in relevant mouse tissues using the BioGPS database^47^. While *Npy* and *Npy1r* are highly transcribed in the brain, they have a wider expression pattern than *Npy2r* and *Npy5r*, although all are more brain specific than *Npy*4*r* and *Npy6r* (Fig. 3A).

To investigate which of the brain specific NPY components (*Npy*, *Npy1*/2/*5r*) may interact with EGR1, we used chromatin immunoprecipitation sequencing (ChIP-seq; generated in a different experiment) to identify potential binding sites for EGR1 in the mouse brain (dorso/ventrolateral striatum). The results demonstrated that *Npy* and *Npy1r* are enriched with EGR1 binding, but not *Npy2r* and *Npy5r* (Fig. 3B). This would predict that, if EGR1 drives transcriptional changes in the NPY system following acute stress, it would likely target *Npy* and *Npy1r*.

Targeted qPCR revealed a sexually dimorphic expression of *Npy* and its receptors in both amygdala and hypothalamus (Table 2). Regarding *Npy*, sex affected these brain regions in opposite directions, where females had more *Npy* expression then males in hypothalamus, while males had more in amygdala (Fig. 3C). Furthermore, as predicted by EGR1 involvement, only *Npy* and *Npy1r* were affected by perinatal LPD (Table 2). More specifically, perinatal LPD led to more *Npy* expression in the hypothalamus, and a weak interaction between perinatal LPD and sex in the amygdala (Table 2). Closer examination revealed that this interaction was mainly due to a sex difference in controls that was absent in animals exposed to perinatal LPD (Fig. 3C).

**Table 2:** Gene expression difference from qPCR in Npy-related genes.

Transcript	Brain region	SEX		LPD		Interaction
		Males vs Females		LPD vs Control		LPD × SEX
		Direction	p-value	Direction	p-value	p-value
Npy	Amygdala	Up	<0.01^a^			
	Hypothalamus	**Down**	**<0.01** ^**b**^	**Up**	**<0.05** ^**f**^	
Npy1r	Amygdala	**Up**	**<0.01** ^**c**^			**<0.1**
	Hypothalamus					
Npy2r	Amygdala					
	Hypothalamus	Up	<0.01^d^			
Npy5r	Amygdala					
	Hypothalamus	Up	<0.01^e^			

Two-way generalized linear models were used resulting in these estimated marginal means based on fold changes:

^a^Males (n = 23): 1.26+/−0.06; Females (n = 24): 1.03+/−0.06. ^b^Males (n = 22): 0.93+/−0.10; Females (n = 24): 1.32+/−0.09. ^c^Males (n = 23): 1.18+/−0.04; Females (n = 24): 1.03+/−0.04. ^d^Males (n = 22): 1.24+/−0.05; Females (n = 23): 1.01+/−0.05. ^e^Males (n = 22): 1.21+/−0.04; Females (n = 21): 1.01+/−0.05. ^f^LPD (n = 26): 1.27+/−0.09; Control (n = 20): 0.99+/−0.1.

### DNA-Methylation in Npy1r amygdala

**Figure 4:**
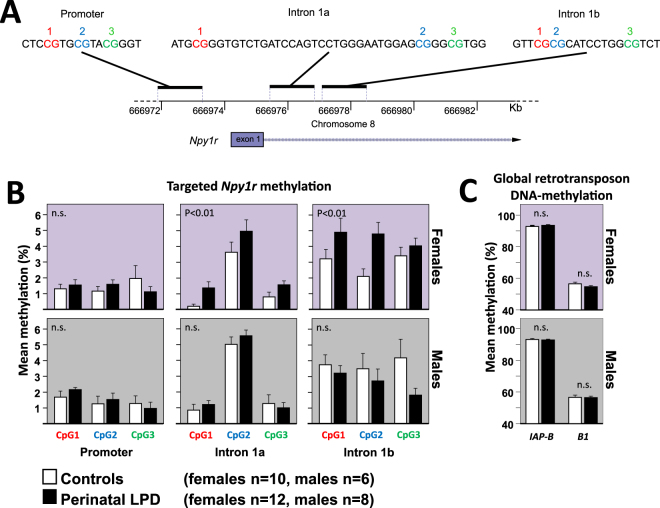
In amygdala, perinatal LPD affects DNA-methylation in the first intron of the *Npy1r* gene in females, but not in males. Three target regions in the Npy1r gene were successfully assayed using BS pyro-sequencing: one in the promoter and two in the first intron (**A**). All assays showed low levels of DNA-methylation (**B**). Since levels of DNA-methylation at neighboring CpGs are never fully independent, a repeated measure Mixed linear model was applied to test all CpGs within a given assay. Statistical interactions between Sex and Perinatal LPD were observed in the Intron 1a and 1b assays, but not in the promoter (see main text). This was primarily due to effects in adult females, in which females of mothers exposed to LPD showed more DNA-methylation than controls (p-values within graphs). There were no global changes in retrotransposon methylation (IAP-B and B1) in adult offspring following perinatal LPD (**C**).

Since perinatal LPD increased the basal level of *Egr1* in the amygdala, and since its effector gene, *Npy1r*, showed sexually dimorphic expression, we asked if epigenetic regulation of EGR1 binding sites in *Npy1r* could explain some of these sex differences. Using bisulfite pyro-sequencing, three regions covering parts of the promoter and the first intron were successfully assayed (Fig. 4A). Results showed interactions between perinatal LPD and Sex on CpG methylation in the first intron of *Npy1r* (Mixed linear model; Intron 1a: Perinatal LPD F = 6.2, p < 0.05, Perinatal LPD x Sex F = 3.7, p < 0.1; Intron 1b: Perinatal LPD x Sex: F = 12.5, p < 0.001), but no effect in the promoter region. More specifically, the effects in the first intron were mainly due to increased methylation in females exposed to perinatal LPD compared to controls (Fig. 4B). This sex-dependent methylation pattern was unaffected by global changes in DNA-methylation, as measured by retrotransposon DNA-methylation (Fig. 4C).

## Discussion

Here, we present a novel link between epigenetic reprogramming by early life adversity, in the form of perinatal low protein diet (LPD), and NPY-dependent stress-regulation in the rodent brain. Our data suggests that sex affects the consequence of perinatal LPD on multiple levels. First, we showed that males exposed to perinatal LPD appeared both more anxious- and depressed-like than controls, while no or opposite relationships were observed in females. Furthermore, several immediate early gene (IEG) transcription factors were differentially expressed either between the sexes or by maternal diet, in which the members of the early growth response family (*Egr1*/*2*/4) were strongly upregulated in both males and females exposed to perinatal LPD. Lastly, we followed the protein of one of these genes, EGR1, to its binding sites in NPY-related genes, and showed sex-specific gene regulatory and epigenetic changes following perinatal LPD.

As expected, the list of genes that were rapidly activated or repressed within the amygdala in response to 15 min of forced swimming contained several well-known immediate early transcription factors, such as *Egr1*/2/4, *Fos*, *FosB*, and *Npas4*. However, novel targets were also identified. The epigenetic regulators *Baz*2*a* and *Smarcad1* ^48^ are of particular importance since they may be involved in the dramatic changes in heterochromatin previously observed in response to acute and chronic stress^49–51^, which constitutes a relatively unexplored path in the etiology of mental disorders^52^.

Early growth response genes (*Egr1*/*2*/*4*) showed consistent upregulation following perinatal LPD. This gene family has five members, *Egr1*/*2*/3/4 and *Wilms’ tumor gene* (*Wt1*). Besides their DNA-binding capability, some members are known to have multiple domains which allow them to both repress and activate transcription of target genes depending on the genomic context^35^. All members but *Wt1* have been implicated in brain development, where *Egr1* has been implicated in memory formation during fear conditioning^53–55^ and spatial learning^56–58^. We show that perinatal LPD in both males and females increases the basal transcription of *Egr1*, while leaving *Fos* and *Npas* gene families unaffected. We also show that this is similar to what happens in fear conditioning, thus may reflect a state that predisposes fear learning in the amygdala. Contrary to c-FOS, which is primarily regarded as a neural activation marker, EGR1 is more specifically considered a marker for neural plasticity^38^. Elevated basal transcription of *Egr1* may therefore indicate ongoing and long-lasting neuronal reorganization in both males and females exposed to perinatal LPD. Since chronic food deprivation has also been shown to increase the basal transcription of *Egr1* ^33^, this may represent a general signature of malnutrition. However, food deprivation also affects other IEGs^33^, and we show that *Egr1* transcription was unaffected by zinc deprivation (Fig. S6). Since perinatal LPD primarily affected the *Egr* family, this indicates that its members may play specific roles in this type of early life programming.

IEG transcription factors showing differential expression between the sexes were consistently downregulated in males (*Cebpd*, *Smarcad1*, *Sox8*, *Faap100*). Downregulation of *Sox8* in males compared to females is unexpected, since it is involved in male embryonic sex determination^59^. Our data indicates that *Sox8* is highly expressed in the adult amygdala in both males and females, which is supported by mouse (GeneAtlas MOE430, gcrma, probeset = 1435438_at), and human (Barcode on normal tissues, probeset = 226913_s_at) datasets reported in BioGPS. This indicates that *Sox8* may have additional roles in the adult brain.

There were only weak statistical interactions between sex and perinatal LPD on IEG expression in amygdala, which indicates that the immediate early response may only play a limited role in controlling the sexually-dimorphic behaviors observed following perinatal LPD. This conclusion must be considered in relation to the limited number of animals included in the microarray experiment. Increasing the sample size may result in stronger interactions. However, even if the immediate early response is not playing a direct role, IEGs may still be involved through mechanisms that target binding sites of their effector genes.

To explore possible effector genes relevant for nutritional stress, we targeted *Npy* and its receptors in two different brain regions known to regulate stress and food intake: amygdala and hypothalamus. Two of the *Npy*-related genes were affected by perinatal LPD: *Npy* and *Npy1r*, which were also the only two binding EGR1. Together with the behavioral data this suggests a possible link between perinatal LPD, changes in *Egr*-family gene regulation, and Npy-related control of anxiety.

Our data also points to a possible explanation for the sexually dimorphic behavioral effects of perinatal LPD. Increased hypothalamic *Npy* expression in perinatal LPD females, and decreased *Npy1r* amygdala expression in LPD males could to some extent explain the different behavioral consequences of perinatal LPD between the sexes. However, two important questions need to be addressed. [i] While hypothalamic NPY may have some anxiolytic effects, the main anxiolytic effect of NPY is believed to be exerted by the activation of NPY1r receptors in the amygdala^24, 25^. So why do perinatal LPD females, which display an anxiolytic behavioral profile compared to perinatal LPD males, have comparatively low expression of both *Npy* and *Npy1r* in amygdala? [ii] Following perinatal LPD, both males and females elevate their baseline expression of *Egr1*. This predicts similar regulatory effects on target effector genes in both sexes. So why do males and females differ in amygdala *Npy1r* expression following perinatal LPD?

**Figure 5:**
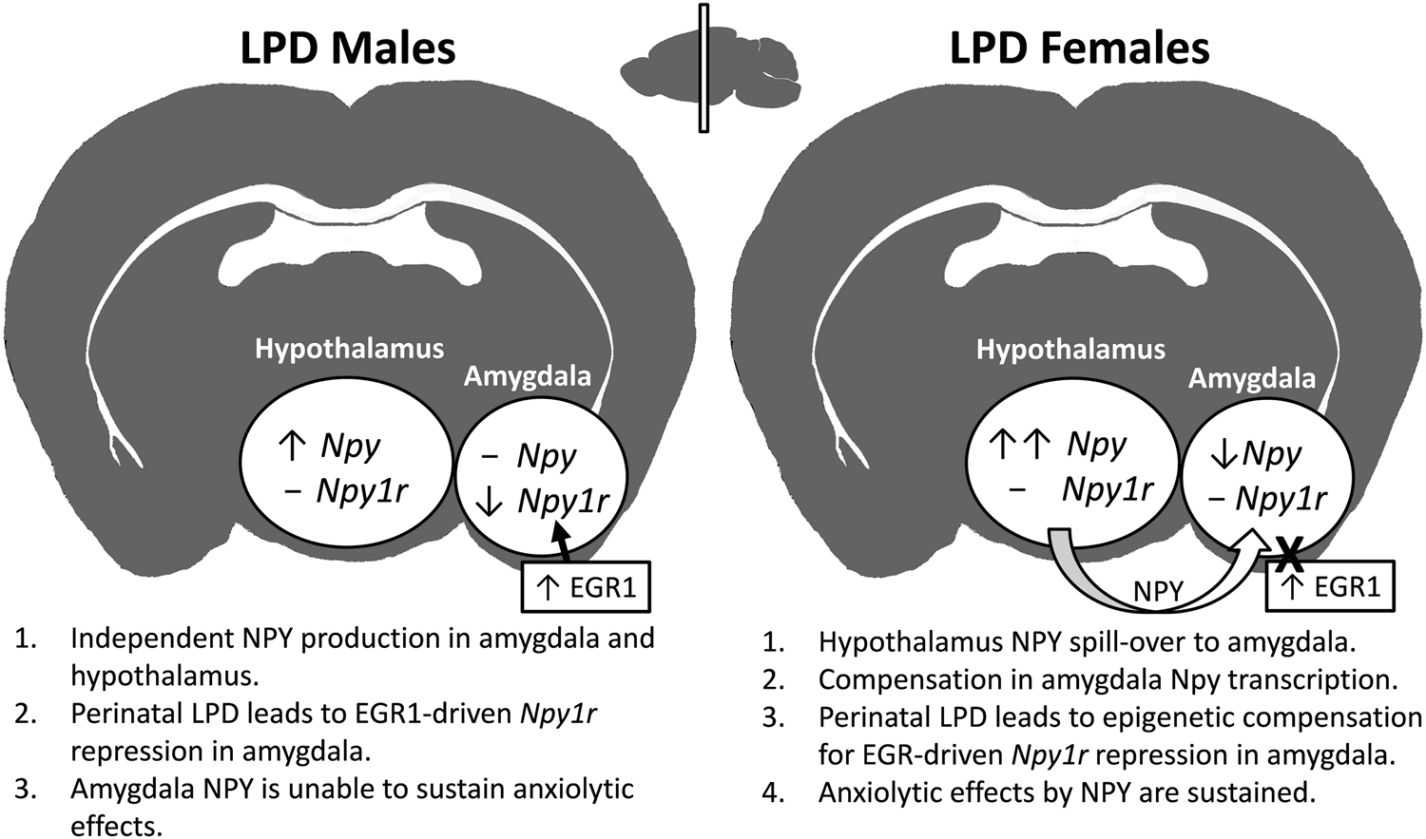
A possible model explaining sexually-dimorphic effects by perinatal LPD in adult offspring. Perinatal LPD leads to different, and sometimes opposite, depression- and anxiety-like behavioral profiles between the sexes, in which males seem vulnerable and females protected against this adversity. Previous studies associate increased NPY1r receptor activation in the amygdala with anxiolytic behavioral profiles. In males exposed to perinatal LPD, high basal transcription of *Egr1* causes down regulation of its effector gene, *Npy1r*, in the amygdala that is consistent with an anxiogenic, vulnerable, phenotype. Our data suggest that Perinatal LPD females are protected from this effect by two mechanisms: [i] by a surplus of NPY in hypothalamus that may spills-over through distal neural projections causing increased levels of NPY in amygdala; [ii] by epigenetic blocking/counteracting mechanisms in the first intron of the *Npy1r* gene, which results in stable expression of *Npy1r* despite repressive pressures by elevated amygdala expression of *Egr1*.

Regarding [i], low female expression of both *Npy* and *Npy1r* in the amygdala predicts higher anxiety only if NPY originates locally within this brain region. Since there are known NPY projections from hypothalamus to the amygdala^26, 27^, it is possible that very high expression of hypothalamic NPY, as observed in females, may “spill-over” to the amygdala. Such spill-over would likely be compensated by lower NPY expression in the amygdala, which is indeed observed in females compared to males. This model predicts that perinatal LPD females, which present the highest *Npy* expression in the hypothalamus, and maintain their amygdala *Npy* and *Npy1r* expression, would show an anxiolytic profile compared to controls with lower hypothalamic *Npy* expression (Fig. 5).

Regarding [ii], since EGR1 may both activate and repress various target genes^35^, it is challenging to predict the consequences following elevated basal *Egr1* expression. However, exposure to perinatal LPD in males is associated with a decrease in *Npy1r* expression in amygdala. Knowing that EGR1 binds the promoter and therefore likely regulates *Npy1r* in this brain region suggests that EGR1 has repressive effects on the expression of *Npy1r*. So why do females not experience the same repression following perinatal LPD? Our data suggest that females compensate using epigenetic mechanisms (Fig. 5). By methylating the first intron of *Npy1r*, perinatal LPD females may either prevent EGR1 from binding to the gene, or they compensate for EGR1 repression by an intragenic gene activation mechanism^60, 61^. In any case, these epigenetic changes would result in stable *Npy1r* expression despite elevated *Egr1* expression. Together with elevated NPY in the amygdala, suggestively caused by hypothalamic spill-over, these molecular factors may confer a protective effect in females exposed to perinatal LPD.

From our perspective, the hypothalamic spill-over model is the best fit for our results (Fig. 5). However, we are aware that this is speculative and in need of further validation in future studies. Of importance is to validate the plausible sexually dimorphic neural projections which transports NPY from hypothalamus to the amygdala. This could be accomplished by using antero- or retrograde tracing to compare the sexes on hypothalamus originated NPY in the amygdala. Gonadectomy may also affect this spill-over, especially since abolishing ovarian hormones in females seems to affect stress-induced gene expression of *Egr1*, *Egr3, and Fos*, as well as *Npy*, in the hypothalamus^62^. Furthermore, while we focused on behavioral stress-responses and *Npy* regulation in the amygdala, the strongest effect by perinatal LPD was seen on *Npy* regulation in the hypothalamus. Future studies must therefore investigate the relationship between the orexigenic and anxiolytic effects of perinatal LPD, and if this is associated with compensatory epigenetic changes in *Npy* and its receptor genes in hypothalamus. In addition, recent findings that early life malnutrition specifically affects regulation of mitochondrial DNA^63^ in a sex typical manner^64^, needs to be investigated in relation to possible neurodevelopmental and behavioral impacts.

Sex bias has repeatedly been identified as a major limitation in medical research^65, 66^, where the majority of studies especially in neuroscience have been performed exclusively on males^67^. This is of particular concern in animal models of human stress-dependent mental disorders, such as major depression and substance use disorders, which show substantial sex differences in patients, regarding both frequency and etiology^68–70^. Our study is a rare attempt to consistently compare males and females by their specific changes at multiple phenotypic levels (behavior, epigenetic, gene expression) following a known risk factor for mental disorders (perinatal malnutrition). Our results indicate a complexity in the relationship between these phenotypic levels that may have many interpretations and mechanistic bases. However, the lack of satisfying explanatory models for many stress-related mental disorders indicates that this molecular complexity is a common feature also in human patients. Therefore, understanding the mechanism behind the relatively static sex differences induced by early adversity in rodents is an important strategy for understanding sex-biased mental disorders in humans.

## Materials and Methods

### Animal husbandry

Sv129 mice were used (males and females; age 7-8 weeks at start of experiments; Jackson Laboratories, ME, USA) and kept under the following conditions: 12:12 h dark/light-cycle (lights on at 6 am); 23 ± 2 °C; 60 ± 10% humidity. Mating was initiated at week 9, with the first day of pregnancy set to the presence of a vaginal plug. After a vaginal plug was noted, males were removed, and dams set on either a low protein diet with 8% protein content (78% carbohydrates; 5% fat) or regular chow with 20% protein content (66% carbohydrates; 5% fat) (Research Diets, NJ, USA^71, 72^). The diet was maintained throughout pregnancy and lactation, i.e. until weaning of the pups. Following weaning (postnatal day 21), male and female pups were separated, group-housed with littermates (max 4 per cage) and maintained on standard laboratory chow with water available *ad libitum*. Experiments were approved by and carried out in accordance with recommendations from Animal Care and Use Committee (ACUC) at the National Institute of Health, Bethesda, MD, USA.

### Behavioral tests

Offspring behavioral evaluation started at 10 weeks of age and was performed between 10 am-3 pm in the light phase. Offspring were weighed at birth and then once per week until end of the experiments. Behavioral tests were spaced at least 72 hrs apart and performed in the order presented below. Procedures for hot plate, and tail flick are described in the Supplemental Material.

### Elevated plus maze

The elevated plus-maze (EPM) measures anxiety-related behavior in rodents^41^. We used an apparatus in black plastic with two open arms (30 × 5 cm) with a small raised lip (0.5 cm) around the edges, and two closed arms (30 × 5 × 15 cm) that extended from a common central platform (5 × 5 cm). Testing was performed under white light (~150 lux) using manual observation (treatment blind). Time in and entries into the open and closed arms were measured, and given as percent time of the total time spent in any arm. Activity was assessed as the number of entries into the closed arms^73^.

### Forced swim

Measures depression-like behavior in rodents. Mice were placed in a cylinder, 20 cm in diameter, filled to a depth of 12 cm with tap water, maintained at 25 ± 2 °C^74^. In the single-trial version of the task, the mouse was observed for the last 4 min of a 6 min trial.

### Open field behavior

Locomotor activity and anxiety-related behavior were assessed in automated open field arenas with photobeam grids (Med Associates Inc., Fairfax, VT, USA). An animal was placed in the middle of the arena (27.3 × 27.3 cm) and allowed to explore for 30 min. Activity in center and periphery, number of crossings between center and periphery, as well as total activity were measured by number of beam breaks.

### Tissue collection and nucleic acid extraction

Whole brains were frozen in −80 °C isopentane after cervical dislocation. Immediately before culling, half of the animals in each group were stressed by means of a prolonged FST (15 min) in moderately tempered water (20 ± 1 °C). Brains were later sliced using a cryostat (Leica 3500 M) and 1 mm micro-punches were pooled for amygdala and hypothalamus respectively according to *The Mouse Brain in Stereotaxic Coordinates* ^75^. DNA and RNA were extracted using the AllPrep DNA/RNA Micro Kit (Qiagen N.V, Venlo, Netherlands) according to manufacturer’s recommendations. All RNA samples showed RNA integrity scores > 8 (Bioanalyzer, Agilent technologies Inc., CA, USA).

### Gene expression microarray analysis in amygdala

Each amygdala RNA sample were pooled in equal proportions with a corresponding sample within each subgroup (e.g. one pool containing two male [sex] controls [maternal diet] exposed to forced swim [stress treatment]). Pools were diluted to 21.7 ng/μl. In total, 16 arrays (32 animals) were used in the original experiment giving 4 arrays within each sex, maternal diet and stress treatment using SurePrint G3 Mouse Exon 4 × 180 K Microarrays (Agilent technologies Inc., CA, USA, design ID: 030493). Labeling, hybridization and scanning was performed according to manufacturer’s protocol (Agilent technologies Inc., CA, USA, Design ID: 030493). A technical replication/validation experiment was performed on the male samples (8 arrays/16 animals) using the same protocol but different microarrays, hybridization oven and scanner (same models, different lab). Signal intensity values were acquired from the Feature Extraction software (v.10.7.3) using default settings (Agilent technologies Inc., CA, USA) and imported into R/Bioconductor, where preprocessing (RMA normalization, followed by low signal probe filtering) and differential expression analysis, were done in limma^76^. A principal component analysis (PCA) revealed a strong first component in the microarray data explained by technical variation (PC1). Two types of factorial linear models were therefore applied on log2 transformed probe values including the following factors: [i] Sex + Maternal diet + Forced swimming, and [ii] PC1 + Sex + Maternal diet + Forced swimming, where PC1 represented factor scores of PC1 extracted from the PCA. Only probes found in the top 1000 differentially expressed (ranked by Bayesian b-value) of both models were considered (Fig. S3A). Of these, only probes that at least tended to be differentially expressed (p < 0.1) in the same direction (represented by log2 fold changes) in the male replication experiment was considered differentially expressed between non-stressed and forced swim exposed animals (Fig. S3B). Array data has been deposited at ArrayExpress (Accession number: E-MTAB-5496; https://www.ebi.ac.uk/arrayexpress/).

### Quantitative PCR in amygdala and hypothalamus

Eighty ng of total RNA was converted to cDNA using the High Capacity cDNA reverse transcriptase kit (ThermoFisher Scientific, MA, USA). Quantitative PCR was set up on a Biomek 2000 robot (Beckman Coulter Inc, CA, USA) and performed on a 7900HT Fast Real Time PCR (Applied Biosystems, CA, USA) using TaqMan® Universal Master Mix and commercial assays (ThermoFisher Scientific, MA, USA). Delta Ct values were normalized to two housekeeping genes (Gapdh: Mm99999915 g1, Actb: Mm00607939 s1 or Ywhaz: Mm03950126_s1). All primers are found in Table S5.

### Bisulfite pyro-sequencing in amygdala

As previously described^51^, amygdala DNA-methylation was measured by bisulfite pyro-sequencing on a Q96 MD pyro-sequencer using the DNA EpiTect Fast DNA Bisulfite Kit and PyroMark PCR Kit (Qiagen N.V, Venlo, Netherlands). Three assays, one in the promoter, and two in the first intron were successfully designed using PyroMark Assay Design software (v2.0; Qiagen N.V, Venlo, Netherlands) and validated to give specific PCR products. Assays for global methylation was also carried out using two assays targeting B1 and IAP retrotransposons that shows high copy numbers. Primers for the B1 assay was adopted from a previous study^77^, while the IAP assay was designed with primers annealing in the repeat region of GeneBank clone M17551.1. All primers are found in Table S5.

### EGR1 tissue ChIP-seq

Brains from eighteen adult C57BL/6 J(JAX) male mice (13-14 weeks old) (approved and in accordance with ethical permit AC-AAAH5804 Y1 M00 at Columbia University, New York, USA), were collected as described above. 1.0 mm micro-punches were taken from the *ventrolateral* and *dorsolateral* part of *striatum*, *rostral* to the *hippocampus*, excluding the *lateral septal nucleus*, but included *nuclueus accumbens* and most of *caudate putamen*; the main structures containing D1/2 dopamine receptors in *striatum* (http://www.brain-map.org/). Similar chromatin immunoprecipitation protocols have been described previously^78, 79^ but here we applied it on individual animals. Frozen punches were crosslinked in 1% fresh formaldehyde with a protease inhibitor (pi) containing PBS buffer, and quenched after 12 min with 0.125 M glycine. After extensive washes in ice cold pi-PBS, punches were homogenized in pi-SDS lysis buffer (1%) by aspirating the tissue 15 times through a 22 gauge syringe needle. Chromatin was fragmented using an ice chilled Bioruptor standard (Diagenode s.a., Seraing, Belgium) in 9 × 5 min cycles (30 s on 30 s off; high power). Immunoprecipitation (IP) was carried out at 4 °C overnight in diluted pi-SDS/Triton X-100 buffer using the Egr-1 Antibody (C-19) X (Santa Cruz Biotechnology, TX, USA) bound to Dynabeads M-280 Sheep anti-Rabbit IgG (Life technologies, CA,USA). IP samples were washed extensively in ice-cold Hepes/LiCl RIPA buffer on a magnetic rack, with a final wash in salty 1xTE buffer (with 50 mM NaCl), before elution in SDS buffer. Release and decrosslinking was done by adding RNase A (1 h, 37 °C) and Proteinase K (2 h, 55 °C), followed by QiaQuick purification (Qiagen N.V, Venlo, Netherlands). Input samples were treated identically to IP samples but with no immunoprecipitation and was pooled prior to library preparation. Sequencing libraries were prepared according to manufacturer’s protocol using NEBNext Ultra™ DNA Library Prep Kit with NEBNext Multiplex Oligos for Illumina (New England Biolabs, MA, USA). Library fragment sizes and concentrations were checked using High sensitivity DNA BioAnalyzer chips (Agilent technologies Inc., CA, USA). The barcoded libraries were diluted to same concentrations, pooled, size selected on an agaros gel (mean fragment sizes of 350 + /−150bp) and purified using QiaQuick (Qiagen, Venlo, Netherlands). The library pool was again assessed on a BioAnalyzer, diluted and sequenced on a NextSeq 500 (Illumina, CA, USA) using 75 bp single end reads, to a mean IP sequencing depth of 19.9 (max = 35.7, min = 14.6), and input pool depth of 37.7 million aligned reads. Reads were aligned to the mm10 mouse genome using BWA mem^80^, and peak calling was done using macs2^81^. True peaks in Npy-related genes were defined as overlapping macs2 calls in at least 14 of 18 samples, and no simultaneous overlap with any input peak. For graph visualization, reads from all samples were pooled. Sequence data for the relevant regions has been deposited at ArrayExpress (Accession number: E-MTAB-5503; https://www.ebi.ac.uk/arrayexpress/).

### Bioinformatics and statistics

Tissue expression data for Npy related genes was downloaded from BioGPS^47^, and heatmaps were constructed using the Log2 transformed values, either directly with raw max and means (Absolute) or by dividing transcript tissue values by the maximum tissue value of each transcript (Relative). Gene ontology analysis was carried out in Webgestalt^82^.

For immediate early transcription factor microarray gene expression, Npy/Npy1/2//5r qPCR gene expression, and Npy1r DNA-methylation analysis, generalized linear models (normal, identity) were used, with Sex and Maternal Diet as main effects, and Forced swimming as a co-factor (with two-way interactions between all factors). To make the statistical models as unified as possible between the microarray experiment (based on pools of individual animals across litters), and the other experiments (based on individual animals), we did not control for maternal/litter attributes (e.g. maternal behavior and litter sizes) in the presented analyses. However, litter was introduced as a nominal co-factor in separate analyses of the behavioral, qPCR and DNA-methylation data, which gave no apparent divergences from the p-values of the original models (data not shown). Thus maternal/litter attributes seemed to have played a limited role in the present experiment.

Contrast microarray datasets, used for validating our findings, were downloaded from Gene Expression Omnibus, and processed the same way as the main gene expression microarray analysis. Dataset GSE74002^42^ was used to verify our full list of IEGs following Forced Swimming, but instead following other acute stressors (foot shock, restraint) and fear conditioning. Based on this comparison, we extracted data from *Egr1*, *Egr*2, *FosB* and *Blm* from GSE76108^45^ and GSE60676^46^ to also validate our top results against effects following a zinc restricted diet and chronic alcohol exposure, respectively. For more information, see Supplemental Material.

## Electronic supplementary material

Supplemental Material
