# Supplementary material for: Perinatal Malnutrition Leads to Sexually Dimorphic Behavioral Responses with Associated Epigenetic Changes in the Mouse Brain
Source: Sci Rep. 2017 Sep 11;7:11082. doi: 10.1038/s41598-017-10803-2 (PMC5593991; doi:10.1038/s41598-017-10803-2)
Supplement: Supplementary file 1 — Supplemental Material [file 41598_2017_10803_MOESM1_ESM.pdf]

Supplementary information

Perinatal Malnutrition Leads to Sexually Dimorphic Behavioral Responses and Associated Epigenetic Changes in the Mouse Brain

Daniel Nätt[1,2]*, Riccardo Barchiesi[1], Josef Murad[1], Jian Feng[3,4], Eric J. Nestler[4], Frances Champagne[2], and Annika Thorsell[1]

Content:

Fig. S1 Tail flick pain response. Page 2
Fig. S2 Hot plate pain response. Page 3
Fig. S3 Validation of transcripts differentially expressed. Page 4
Fig. S4 Egr1 technical replication in males. Page 5
Fig. S5 Effects on immediate early transcription factors… Page 6

Tab. S1 Birth variables. Page 7
Tab. S2 Offspring health assessment. Page 8
Tab. S3 Validated list of transcripts affected by 15 min forced swim. Page 9-11
Tab. S4 Effect of acutes stress (footshock, restraint) in the amygdala… Page 12
Tab. S5 Primers used in study. Page 13

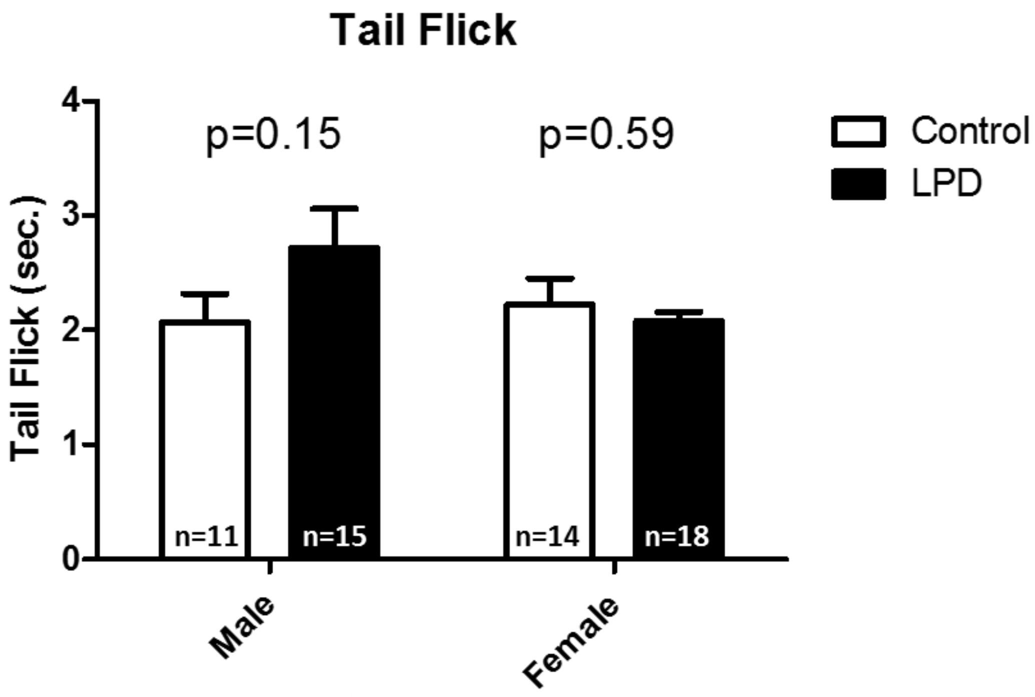

Fig S1. No significant differences in peripheral pain response as measured in the tail flick test could be detected. A trend-level p-value for pain-response in male LPD offspring vs. controls was seen, but it did not reach statistical significance. The tail-flick test (Columbus Instruments, Columbus, OH, USA) assesses spinal response to pain. A mouse's tail were positioned above a heat-source and the time it took for the animal to withdraw it was recorded. Distance from the base of the tail to the heat-source was 4-5 cm and a cut-off time of 8 s was deployed in order to prevent tissue damage.

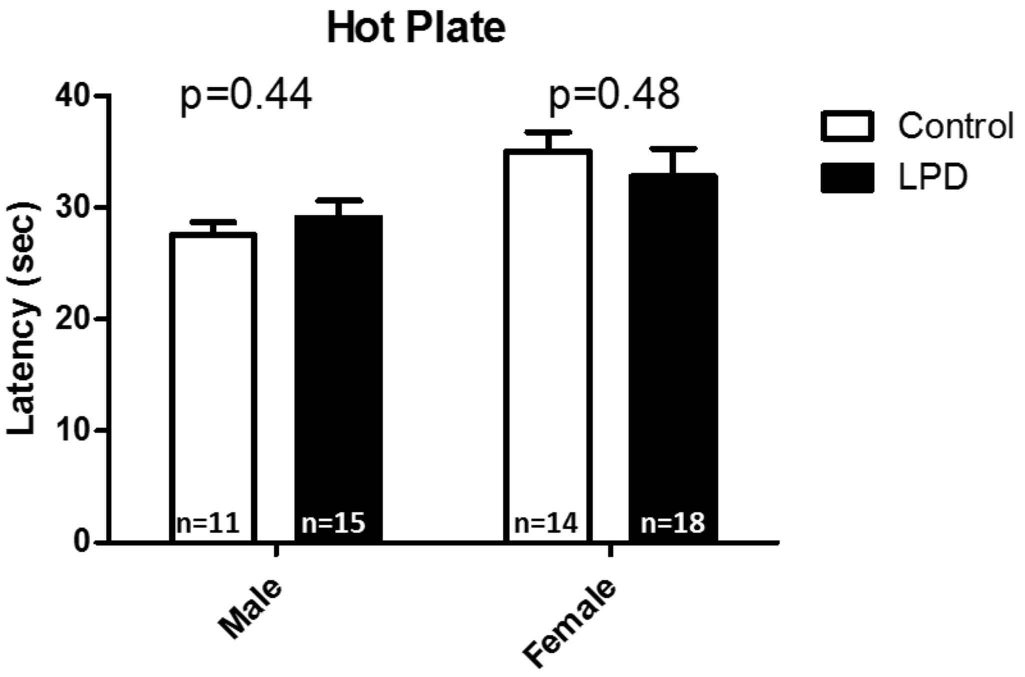

Fig S2. No significant differences in central pain response as measured in the hot plate test could be detected. The hot-plate (Columbus Instruments, Columbus, OH, USA) test assesses spinal and supra-spinal pain pathways. Mice were placed in the middle of a hot plate (55°C ± 0.1°C), and the response latency was measured as the time between placement to the occurrence of nociceptive behavior (licking, shaking of hind paws, or jumping off the surface).

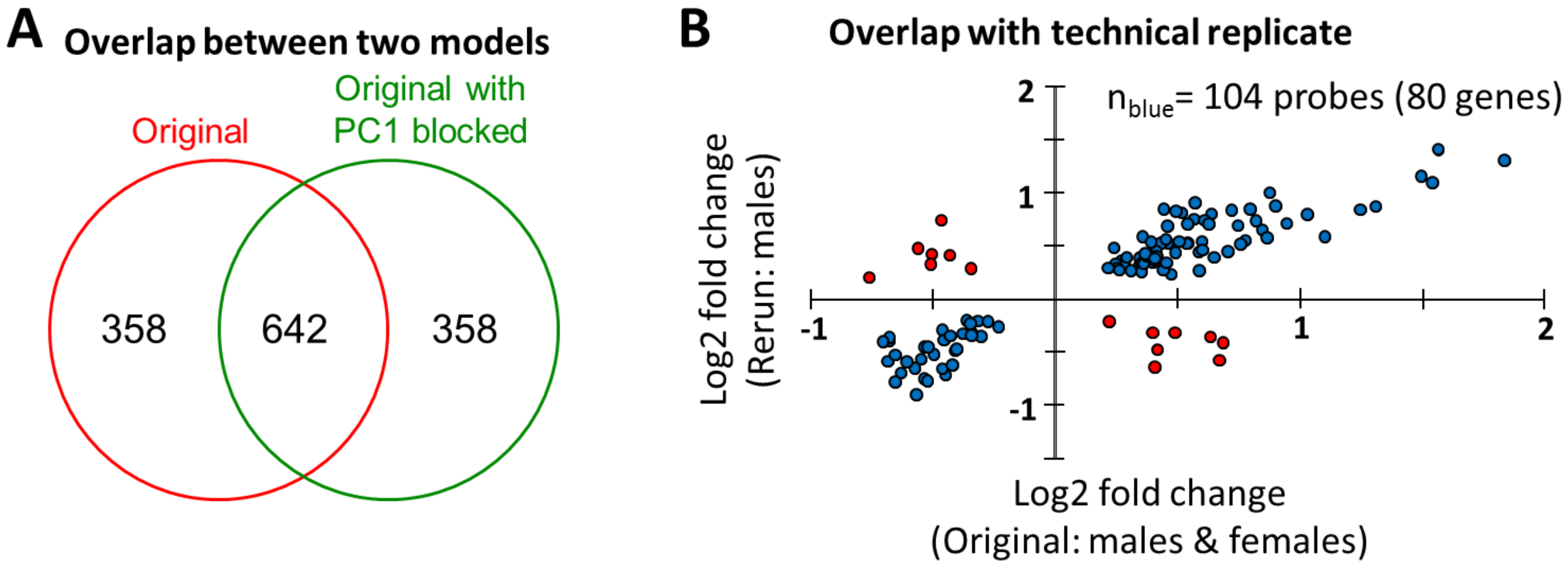

Fig S3. Validation of transcripts (microarray probes) differentially expressed after 15 min forced swim. **A)** Wenn-diagram showing the overlap between the top 1000 differentially expressed probes using two separate statistical models, with or without including the factor scores from the primary factor (PC1; explaining 98% of the variance) generated from the normalized raw signal intensities. **B)** Scatter plot showing the integrity of the 119 probes that were identified in A) and showed a similar effect by acute stress in a technical replication experiment in males ($p<0.1$). The 104 probes that maintained their integrity across statistical models and microarrays (blue dots) were used for further analysis.

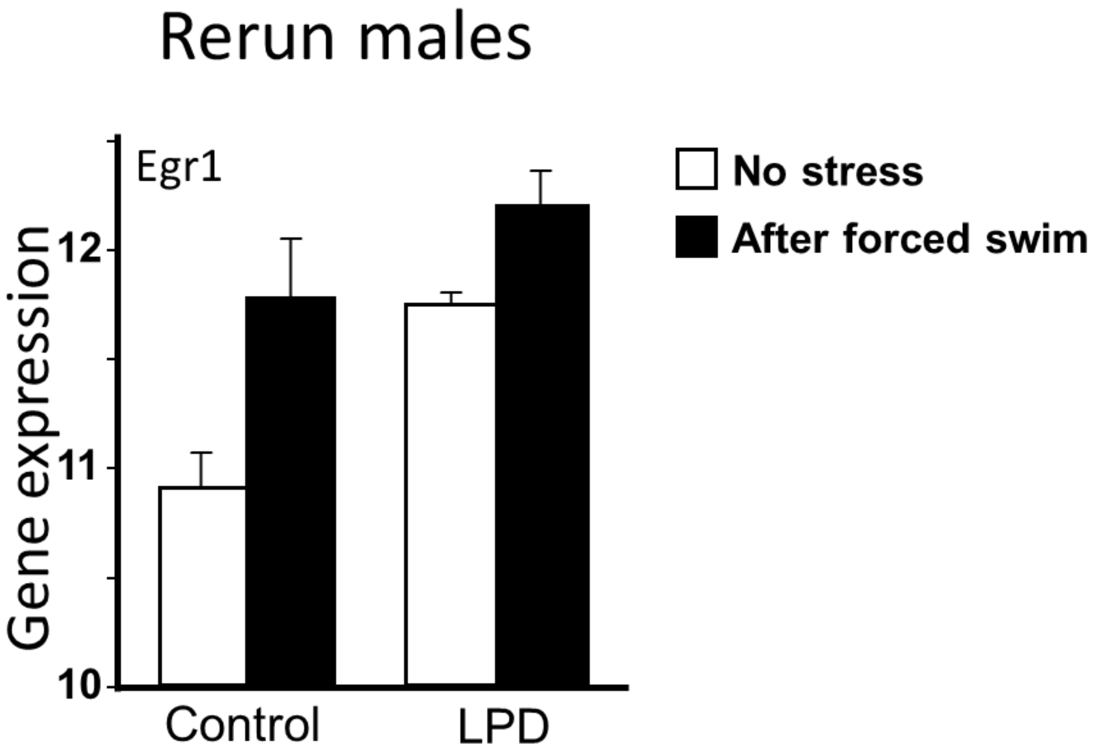

Fig S4. *Egr1* technical replication in males. Similar effects of perinatal LPD on *Egr1* gene expression, as was originally observed in both males and females, was observed when technically replicating the male samples on separate microarrays, using a different microarray scanner. As in the original scan, each array contained a pool of two amygdala/animals; 8 animals per group.

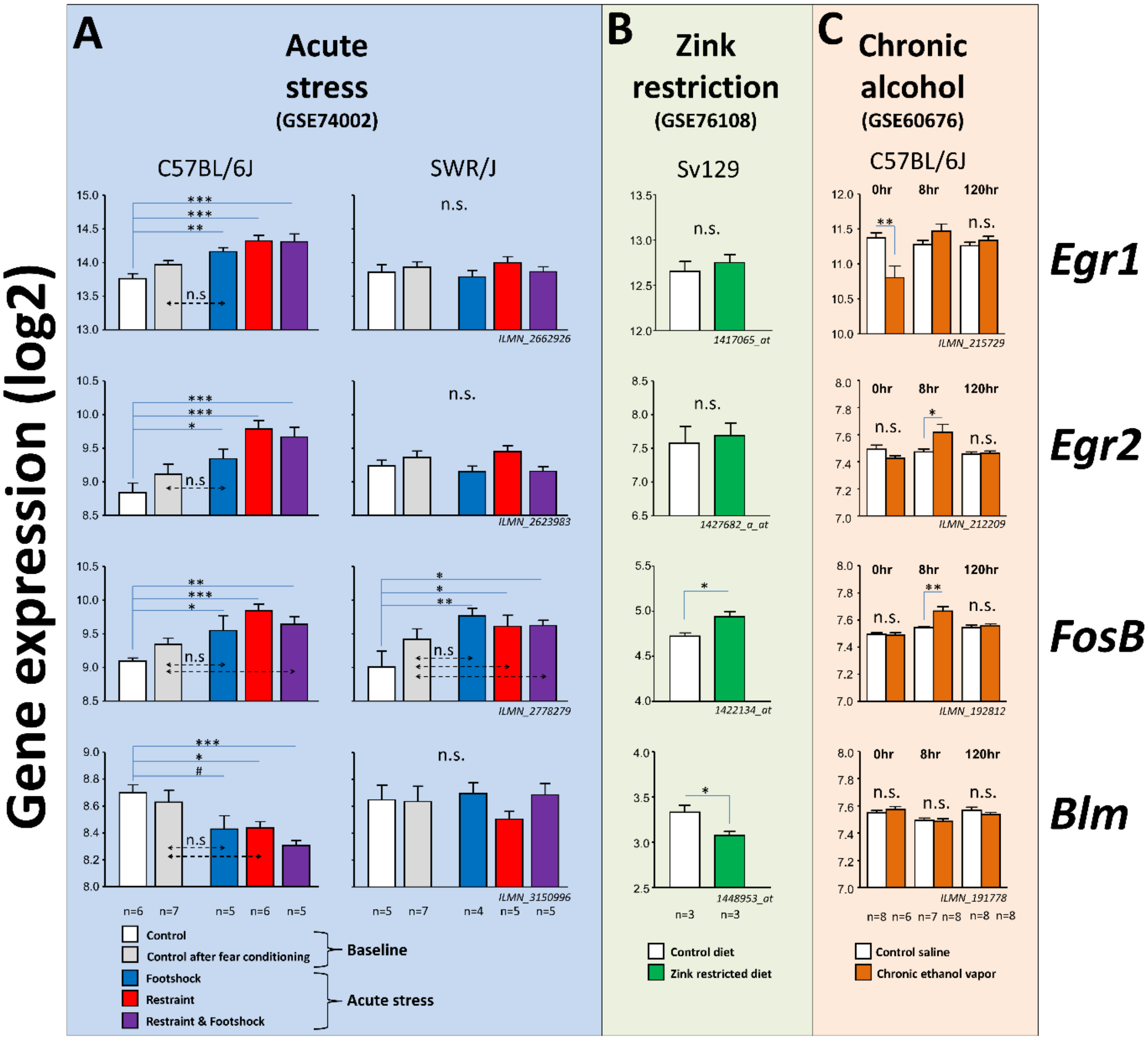

Fig S5. Effects on immediate early transcription factors across studies, microarray platforms and stressors in the amygdala of adult mice. Transcript subset was chosen based on the top overlaps presented in Table S4. Blue panel **A)** shows the effect of different acute stressors and fear conditioning in two mouse strains (C57BL/6J and SWR/J) using data from Szklarczyk *et al.* 2016 (datset:GSE74002; Illumina MouseWG-6 v2 microarrays). Notice that acute stress primarily induced effects in C57BL/6J mice, in which fear conditioning led to increased baseline transcription, much similar to what prenatal LPD induced in the present study. Green panel **B)** shows an effect on *FosB* and *Blm*, but not the *Egr* family, following a zinc restricted diet in Whittle *et al.* 2016 (GSE76108; Affymetrix Mouse Genome 430 2.0 Array) in Sv129 mice. Orange panel **C)** shows the effects of chronic intermittent ethanol exposure in C57BL/6J mice, by four weekly cycles of ethanol vapor exposures, with one week of rest in between, as presented in Osterndorff-Kahanek *et al.* 2015 (GSE60676; Illumina MouseRef-8 v2.0 expression beadchip). Notice that the *Egr* family seems to downregulate immediately following four weekly cycles of ethanol exposures (0h), which signifies the relief/sedative phase of alcohol addiction. The same genes as well as *FosB* are upregulated 8h after vapor exposure, which overlaps the withdrawal/stress phase. All effects are abolished after 120h, which may represent a baseline. Statistical significance are indicated by: # $p<0.1$, * $p<0.05$, ** $p<0.01$, *** $p<0.001$, **** $p<0.0001$.

Table S1. Birth variables.

	Control diet		*Low protein diet*	
Number of litters (n)	9		10	
Successful pregancies (%)	75.0		83.3	
Litter size at birth (n)[a]	4.3	±0.7	3.8	± 0.7
Body-weight dam at birth (g)[a]	23.3	±0.8	21.9	± 0.9
Body-weight dam at weaning (g)	26.4	±1.2	24.8	± 1.1

a =“at birth” indicates in the morning the night after the pups were born.

*p<0.05 vs. control. Data are given as mean ± SEM.

Table S2. Offspring health assessment.

Behavioral assessment	***Control diet***				***Low Protein Diet***			
	Females		***Males***		***Females***		***Males***	
Appearance[a]	3.0	±0.0	3.0	±0.0	3.0	±0.0	3.0	±0.0
Novel environment[b]	1.1	±0.1	1.0	±0.3	1.3	±0.4	1.9	±0.3*
Approach/avoidance[c]	0.8	±0.3	0.9	±0.2	1.0	±0.2	0.9	±0.1
Reflexes[d]	4.0	±0.1	3.9	±0.2	4.0	±0.1	3.7	±0.4
Grip strength (sec)[e]	58.0	±3.0	56.0	±6.0	55.0	±8.0	59.0	±6.0

Offspring were assessed with regard to overall health and behavior using a testing battery according to Karlsson *et al.* 2005. A score was given for each variable assessed, except grip strength, and locomotor behavior (reported in main text, Fig. 1F) for which absolute measures are available (grams, time in seconds and distance travelled). Both male and female offspring were tested (n=7-11 per group; age 5-6 weeks).

a Scoring general appearance: Bald patches, whiskers present, proper grooming. Normal score=3.

b Behavior in novel environment: Animal is placed in an empty cage for 3 min and assessed for behaviors; wild running, excessive grooming, freezing, and body posture. Normal score=0-1.

c Approach/avoidance: Reaction to cotton-tip swab introduced into the empty cage. Normal score=1.

d Reflexes: The cage is moved rapidly from side-to-side and response observed, righting reflex, eye blink, whisker twitch. Normal score=4.

e Grip strength: Hanging from wire lid.

* $p<0.05$ vs. control. Data are given as mean ± SEM.

Table S3. Validated list of transcripts affected by 15 min forced swim

Gene Symbol	*Probe name*	*GenBank ID*	*Genomic location (chr:start-stop)*	*Log2 Fold Change*	*Mean signal intensity*
4921511H03Rik	A_52_P161052	NM_027603	5:7305077-7305137	0.41	1.67
8430408G22Rik	A_56_P153508	NM_145980	6:116602504-116602564	0.54	7.97
Abca9	A_56_P202011	NM_147220	11:110021713-110021773	0.45	3.45
Abca9	A_56_P202012	NM_147220	11:110020268-110020328	-0.41	4.64
Abcc1	A_56_P084165	NM_008576	16:14461182-14461242	-0.52	4.46
Ada	A_56_P031957	NM_007398	2:163558025-163558085	0.51	3.25
Amy1	A_55_P1983418	NM_007446	3:113258900-113258960	-0.57	6.88
Amy1	A_56_P171644	NM_007446	3:113261240-113261300	-0.53	6.32
Anxa9	A_55_P2092869	NM_001085383	3:95100038-95100098	-0.63	3.87
Apold1	A_56_P154108	NM_001109914	6:134934773-134934833	0.78	6.84
Arc	A_56_P218404	NM_018790	15:74501206-74501266	0.60	5.98
Arl4d	A_55_P2057528	NM_025404	11:101529031-101529091	0.46	8.21
Baz2a	A_56_P175718	NM_054078	10:127559437-127559497	0.26	6.18
Blm	A_56_P039900	NM_007550	7:87639053-87639113	-0.57	4.32
Btg2	A_52_P31543	NM_007570	1:135972093-135972153	0.85	7.44
Cabp5	A_55_P2055504	NM_013877	7:13988673-13988733	0.28	2.20
Ccnf	A_56_P100574	NM_007634	17:24376791-24376851	-0.61	4.20
Cd3g	A_56_P135326	NM_009850	9:44782297-44782357	0.75	2.36
Cebpd	A_51_P444447	NM_007679	16:15888458-15888518	0.54	8.74
Cfap61	A_56_P021605	NM_175280	2:145765667-145765727	-0.52	4.36
Creb3l1	A_56_P029674	NM_011957	2:91842146-91842206	0.57	2.30
Crybg3	A_56_P087240	NM_174848	16:59502974-59503034	0.41	2.77
Cyr61	A_56_P170231	NM_010516	3:145311160-145311220	0.95	5.42
Cyr61	A_56_P170230	NM_010516	3:145311496-145311556	0.88	4.50
Cyr61	A_56_P170232	NM_010516	3:145310311-145310371	0.90	6.17
Ddit4	A_51_P245796	NM_029083	10:59412485-59412545	0.37	12.85
Ddx60	A_56_P124104	NM_001081215	8:64429322-64429382	0.54	2.68
Dusp1	A_51_P430900	NM_013642	17:26642525-26642536	0.57	9.26
Dusp1	A_56_P106742	NM_013642	17:26645295-26645355	0.60	5.68
Egr1	A_51_P367866	NM_007913	18:35024394-35024454	0.43	11.61
Egr2	A_55_P2033362	NM_010118	10:67004797-67004857	0.82	8.03
Egr4	A_51_P270426	NM_020596	6:85461152-85461212	0.41	11.26
Faap100	A_56_P197467	NM_027980	11:120234420-120234480	-0.55	5.07
Fmnl3	A_56_P216297	NM_011711	15:99156287-99156347	-0.68	3.56
Fos	A_52_P262219	NM_010234	12:86817899-86817959	1.57	8.63
Fos	A_56_P111481	NM_010234	12:86814978-86815038	1.25	5.63

Fos	A_56_P111483	NM_010234	12:86816542-86816602	1.50	5.00
Fos	A_56_P111482	NM_010234	12:86815888-86815948	1.10	6.15
Fosb	A_55_P2113051	NM_008036	7:19888167-19888227	0.27	8.84
Fosb	A_56_P048513	NM_008036	7:19895166-19895226	0.80	3.50
Frem2	A_56_P169944	NM_172862	3:53393638-53393698	-0.46	1.64
Gadd45g	A_51_P315904	NM_011817	13:51943732-51943792	0.35	12.04
Gadd45g	A_56_P093512	NM_011817	13:51942141-51942201	0.42	7.93
Gadd45g	A_56_P093514	NM_011817	13:51943009-51943069	0.42	7.52
Gem	A_55_P2111790	NM_010276	4:11641691-11641751	0.59	5.37
Gm11237	A_56_P011803	NM_001256481	4:73315078-73315138	-0.70	2.74
Gm266	A_55_P2073694	NM_001033248	12:112723474-112723534	-0.36	5.68
Gpr55	A_55_P2087850	NM_001033290	1:87836216-87836276	0.37	1.99
Gtpbp2	A_56_P102169	NM_019581	17:46304278-46304338	-0.32	6.85
Gtpbp3	A_56_P126039	NM_032544	8:74014298-74014358	-0.35	5.85
Hgd	A_56_P086436	NM_013547	16:37628629-37628689	0.46	1.76
Hk3	A_56_P097649	NM_001206391	13:55115787-55115847	0.39	2.64
Hps5	A_56_P039007	NM_001005247	7:54020188-54020248	-0.40	4.59
Iqce	A_56_P058550	NM_028833	5:141141963-141142023	-0.42	4.93
Itga1	A_56_P092151	NM_001033228	13:115770980-115771040	0.71	3.47
Itgb3	A_56_P200439	NM_016780	11:104498468-104498528	0.36	2.62
Kcnab3	A_56_P200510	NM_010599	11:69144977-69145037	-0.30	6.43
Klf2	A_51_P144264	NM_008452	8:74845136-74845196	0.72	8.48
Klf2	A_56_P126775	NM_008452	8:74844059-74844119	0.63	5.10
Klhl40	A_56_P137529	NM_028202	9:121687985-121688045	-0.43	6.21
Lrba	A_56_P170066	NM_001077687	3:86160608-86160668	0.25	6.10
Map3k2	A_56_P118977	NM_011946	18:32356548-32356608	0.24	9.39
Map3k2	A_56_P118980	NM_011946	18:32363481-32363541	0.37	6.05
Mcpt1	A_56_P189800	NM_008570	14:56638380-56638440	0.48	1.76
Mlana	A_56_P004338	NM_029993	19:29781313-29781373	0.44	2.48
Mme	A_56_P170362	NM_008604	3:63151079-63151139	0.76	3.58
Mmp25	A_51_P187262	NM_001033339	17:23767089-23767149	0.38	2.33
Mup1	A_56_P007456	NM_001163010	4:60512137-60512197	-0.65	2.21
Nfkbia	A_56_P112272	NM_010907	12:56590892-56590952	0.29	12.00
Nfkbia	A_56_P112271	NM_010907	12:56591394-56591454	0.25	11.01
Nme8	A_55_P2125801	NM_001167909	13:19752374-19752434	0.50	2.06
Npas4	A_56_P004566	NM_153553	19:4989641-4989701	0.87	6.02
Npas4	A_55_P1975215	NM_153553	19:4984355-4984415	1.55	9.64
Npas4	A_56_P004596	NM_153553	19:4988075-4988135	1.03	5.12
Npas4	A_56_P004595	NM_153553	19:4988476-4988536	0.65	6.91
Npas4	A_56_P004598	NM_153553	19:4987315-4987375	1.31	6.19
Npas4	A_56_P004599	NM_153553	19:4985970-4986030	1.84	3.64
Nusap1	A_56_P033013	NM_133851	2:119461263-119461323	0.46	1.93
Pkp3	A_56_P051210	NM_001162924	7:148268623-148268683	-0.53	3.88

Plb1	A_56_P066222	NM_001081407	5:32657725-32657785	-0.45	5.08
Plekhn1	A_56_P020477	AK048084	4:155599828-155599888	0.31	1.49
Plk5	A_56_P174768	NM_183152	10:79825819-79825879	-0.37	7.57
Ptgs2	A_51_P254855	NM_011198	1:151954743-151954803	0.59	6.59
Rhcg	A_56_P049499	NM_019799	7:86746472-86746532	0.46	2.44
Rps6kb2	A_56_P002886	NM_021485	19:4159027-4159087	-0.50	4.99
Rsrp1	A_56_P010753	NM_023665	4:134482673-134482733	0.40	10.35
Sgk1	A_56_P182596	NM_011361	10:21714571-21714631	0.50	6.38
Sik1	A_55_P2061278	NM_010831	17:31981254-31981314	0.36	7.21
Sik1	A_56_P100609	NM_010831	17:31991152-31991212	0.64	5.79
Sik1	A_56_P100608	NM_010831	17:31991865-31991925	0.52	7.57
Sik1	A_56_P100610	NM_010831	17:31988477-31988537	0.61	6.24
Smarcad1	A_56_P162270	NM_007958	6:65023197-65023257	-0.28	8.35
Sox18	A_56_P034151	NM_009236	2:181405886-181405946	-0.68	3.25
Sox8	A_52_P269672	NM_011447	17:25703093-25703153	0.22	9.10
Spaca1	A_55_P1972659	NM_026293	4:34116045-34116105	-0.34	1.81
Sppl2b	A_56_P174463	NM_175195	10:80324993-80325053	-0.68	4.73
Tiparp	A_56_P173074	NM_178892	3:65356574-65356634	0.36	8.69
Tiparp	A_56_P173072	NM_178892	3:65350237-65350297	0.35	6.88
Tiparp	A_56_P173073	NM_178892	3:65351547-65351607	0.36	7.46
Trem3	A_56_P107483	NM_021407	17:48386943-48387003	-0.46	3.01
Wbp11	A_56_P156624	NM_021714	6:136766451-136766511	-0.23	7.79
Zan	A_56_P059961	NM_011741	5:137866817-137866877	-0.65	3.91
Zfp536	A_56_P049441	NM_172385	7:38353218-38353278	-0.34	5.57
Zfp692	A_56_P209256	NM_182996	11:58123775-58123835	-0.45	5.79

List was validated by:

[i] only considering microarray probes that were represented among the 1000 top differentially expressed probes (sorted by B-value) when analyzing the data both with and without PC1 as covariate (see Fig S3A).

[ii] a microarray experiment technically replicating the original male sub-experiment (see Figure S3B).

Table S4. Effect of acute stress (footshock, restraint) in the amygdala compared to the present study.

	Szklarczyk et al.														Nätt et al.
		**C57BL/6J**						**SWR/J**							*Same direction as after forced swim in present study*
		**Control**		**Acute Stress**				**Control**		**Acute Stress**					
Gene	*Illumina ID*	*Mean*	*SEM*	*Mean*	*SEM*	*F*	*Sig.*	*Mean*	*SEM*	*Mean*	*SEM*	*F*	*Sig.*	Direction	
Sgk1	ILMN_1213954	12.01	0.12	12.78	0.08	29.65	****	11.98	0.11	12.19	0.07	2.78	n.s.	Up	Yes
Egr1	ILMN_2662926	13.76	0.08	14.26	0.05	28.46	****	13.86	0.09	13.89	0.06	0.08	n.s.	Up	Yes
Egr2	ILMN_2623983	8.84	0.14	9.60	0.09	20.98	***	9.23	0.09	9.26	0.06	0.05	n.s.	Up	Yes
Blm	ILMN_3150996	8.70	0.06	8.39	0.04	18.27	***	8.65	0.09	8.62	0.05	0.06	n.s.	Down	Yes
Fosb	ILMN_2778279	9.09	0.12	9.68	0.08	16.30	***	9.01	0.15	9.66	0.09	13.57	**	Up/Up	Yes
Gtpbp2	ILMN_2550240	8.82	0.07	8.72	0.04	1.51	n.s.	7.80	0.09	8.15	0.05	12.05	**	Up	No
Fos	ILMN_2750515	10.25	0.14	10.78	0.09	10.86	**	10.07	0.14	10.19	0.08	0.50	n.s.	Up	Yes
Nfkbia	ILMN_3001914	9.27	0.10	9.60	0.06	8.17	*	9.05	0.11	9.24	0.06	2.49	n.s.	Up	Yes
Hk3	ILMN_3161013	7.75	0.05	7.59	0.03	7.82	*	7.74	0.07	7.63	0.04	2.13	n.s.	Down	No
Klf2	ILMN_2604029	9.34	0.12	9.62	0.07	4.15	#	8.92	0.08	9.17	0.05	7.80	*	Up/Up	Yes
Gm266	ILMN_2989204	8.28	0.06	8.10	0.04	6.72	*	8.14	0.07	8.12	0.04	0.03	n.s.	Down	Yes
Mme	ILMN_2863362	8.06	0.08	8.14	0.05	0.54	n.s.	7.76	0.08	8.00	0.05	6.41	*	Up	Yes
Blm	ILMN_2774640	8.60	0.07	8.40	0.04	6.17	*	8.66	0.09	8.59	0.06	0.33	n.s.	Down	Yes
Trem3	ILMN_2915303	7.39	0.06	7.56	0.04	6.15	*	7.55	0.05	7.43	0.03	4.24	#	Up/Down	-
Rps6kb2	ILMN_2611256	7.67	0.05	7.79	0.03	3.32	#	7.87	0.06	7.70	0.04	6.02	*	Up/Down	-
Creb3l1	ILMN_2535223	7.31	0.03	7.39	0.02	5.94	*	7.33	0.06	7.36	0.03	0.23	n.s.	Up	Yes
Ptgs2	ILMN_1231600	7.51	0.07	7.66	0.04	3.84	#	7.42	0.08	7.64	0.05	5.37	*	Up/Up	Yes
Nfkbia	ILMN_1218605	8.37	0.19	8.87	0.12	5.07	*	8.49	0.21	8.62	0.13	0.27	n.s.	Up	Yes
Plb1	ILMN_3109753	7.36	0.06	7.31	0.04	0.51	n.s.	7.29	0.03	7.37	0.02	4.63	*	Up	No
Egr4	ILMN_1215713	11.07	0.19	11.55	0.12	4.55	*	11.25	0.12	11.38	0.07	0.82	n.s.	Up	Yes
Mup1	ILMN_2875730	7.34	0.30	7.54	0.19	0.32	n.s.	7.53	0.10	7.29	0.06	4.39	#	Down	Yes
Klhl40	ILMN_2636054	8.32	0.06	8.21	0.04	2.33	n.s.	8.35	0.09	8.14	0.05	4.26	#	Down	Yes
Arl4d	ILMN_2593709	7.41	0.06	7.45	0.04	0.30	n.s.	7.70	0.09	7.49	0.05	4.11	#	Down	No
Hgd	ILMN_1212739	7.57	0.05	7.46	0.03	3.88	#	7.55	0.07	7.53	0.04	0.02	n.s.	Down	No
4930529M08Rik	ILMN_2552055	7.47	0.05	7.36	0.03	3.70	#	7.37	0.07	7.38	0.04	0.04	n.s.	Down	No
Cyr61	ILMN_2710253	7.76	0.04	7.68	0.02	3.56	#	7.65	0.08	7.66	0.05	0.03	n.s.	Down	No
Anxa9	ILMN_2715226	7.73	0.04	7.64	0.03	3.44	#	7.87	0.07	7.71	0.04	3.48	#	Down/Down	Yes
Tiparp	ILMN_2589291	7.42	0.04	7.50	0.03	3.02	#	7.44	0.06	7.38	0.04	0.80	n.s.	Up	Yes

Dataserie GSE74002 was downloaded from Gene Expression Omnibus. Data was generated by Szklarczyk *et al.* in a study of acutes stress (groups: footshock, restraint stress or both) and fear conditioning (not included in the present analysis) in two strains of mice (C57BL/6J and SWR/J). Following extraction of all available data (quantile normalized) from transcripts that were significanlty affected by acute force swimming in the presents study, a General Linear Model with Acute stress (either footshook, restraint or both) as the main factor simulatanously controlling for strain (Gene expression = Acute stress + Strain) was carried out. Correction for multiple testing was not conducted since any evidence of overlap between studies was given priority.

Statistical significance are indicated by: # p<0.1, * p<0.05, ** p<0.01, *** p<0.001, **** p<0.0001 .

Table S5. Primers used in study.

	Purpose	Target	Info	Primer type	Sequence / product code
qPCR *					
	Housekeeping gene	Gapdh	TaqMan Gene Expression Assay		Mm99999915_g1
	Housekeeping gene	Actb	TaqMan Gene Expression Assay		Mm00607939_s1
	Housekeeping gene	Ywhaz	TaqMan Gene Expression Assay		Mm03950126_s1
	Target gene expression	Npy	TaqMan Gene Expression Assay		Mm03048253_m1
	Target gene expression	Npy1r	TaqMan Gene Expression Assay		Mm00650798_g1
	Target gene expression	Npy2r	TaqMan Gene Expression Assay		Mm01956783_s1
	Target gene expression	Npy5r	TaqMan Gene Expression Assay		Mm02620267_s1
***BS pyro seq* #**					
	Target DNA-methylation	Npy1r Promoter	Custom assay	Forward primer	AGTTAAGGATTAGTGGGGTGAGAG
				Reverse primer (biotinylated)	TTTCCCCCACAACCTCCTC-Bio
				Sequencing primer	AGTGGGGTGAGAGTT
	Target DNA-methylation	Npy1r Intron 1a	Custom assay	Forward primer	ATTGATGGGGTGAGGTATGA
				Reverse primer (biotinylated)	CCCAAACCTATCCCACAATCTAAATTA-Bio
				Sequencing primer	GTGAGGTATGAGAGG
	Target DNA-methylation	Npy1r Intron 1b	Custom assay	Forward primer	GGATGTGAGATTTTAAAGGGAAAGTG
				Reverse primer (biotinylated)	AAAACACCTCCCCTAACTTC-Bio
				Sequencing primer	GGGAAAGTGTATATTTTTTAGAG
	Global DNA-methylation	B1	Custom assay	Forward primer	TTGGTTATTTTGGAATTTATTTTGTAGAT
				Reverse primer (biotinylated)	TAATAACACACACCTTTAATCCCAACA-Bio
				Sequencing primer	TTTGGAATTTATTTTGTAGATTAG
	Global DNA-methylation	IAP	Custom assay	Forward primer	TGGGTTGTAGTTAATTAGGGAGTGA
				Reverse primer (biotinylated)	AACACCACAAACCAAAATCTTCTA-Bio
				Sequencing primer	GTAGTTAATTAGGGAGTGAT

* PCR conditions for all qPCR assays: 50°C 2 min -> 95°C 10min -> (95°C 15s -> 60°C 1 min) x 40 cycles

PCR conditions for all BS pyro seq assays: 95°C 15 min -> (94°C 30s -> 56°C 30s -> 72°C 30s) x 50 cycles -> 72°C 10 min
